# Multi-platform microRNA profiling of hepatoblastoma patients using formalin fixed paraffin embedded archival samples

**DOI:** 10.1186/s13742-015-0099-9

**Published:** 2015-11-25

**Authors:** Anna L. Leichter, Rachel V. Purcell, Michael J. Sullivan, Michael R. Eccles, Aniruddha Chatterjee

**Affiliations:** 1 Department of Pathology, Dunedin School of Medicine, University of Otago, 270 Great King Street, P.O. Box 913, Dunedin, 9054 New Zealand; 2 Children’s Cancer Research Group, University of Otago, Christchurch, New Zealand; 3 Royal Children’s Hospital, Melbourne, VIC Australia; 4 Maurice Wilkins Centre for Molecular Biodiscovery, Level 2, 3A Symonds Street, Auckland, New Zealand

**Keywords:** miRNA, FFPE, RNA expression, Microarray, Next-generation sequencing, NanoString, Hepatoblastoma, Epigenetics

## Abstract

**Background:**

Formalin fixed paraffin embedded (FFPE) samples are a valuable resource in cancer research and have the potential to be extensively used. However, they are often underused because of degradation and chemical modifications occurring in the RNA that can present obstacles in downstream analysis. In routine medical care, FFPE material is examined and archived, therefore clinical collections of many types of cancers exist. It is beneficial to assess and record the quality of data that can be obtained from this type of material. The current study investigated three independent platforms and their ability to profile microRNAs (miRNAs) within FFPE samples from hepatoblastoma (HB) patients.

**Findings:**

Here we present three types of datasets consisting of miRNA profiles for 13 HB patients with different tumour types and molecular variations. The three platforms that were used to generate these data are: next-generation sequencing (Illumina MiSeq), microarray (Affymetrix^®^ GeneChip^®^ miRNA 3.0 array) and NanoString (nCounter, Human v2 miRNA Assay). The mature miRNAs identified are based on miRBase version 17 and 18.

**Conclusions:**

These datasets provide a global landscape of miRNA expression for a rare childhood cancer that has not previously been well characterised. These data could serve as a resource for future studies aiming to make comparisons of HB miRNA profiles and to document aberrant miRNA expression in this type of cancer.

**Electronic supplementary material:**

The online version of this article (doi:10.1186/s13742-015-0099-9) contains supplementary material, which is available to authorized users.

## Background

MicroRNAs (miRNAs) are a large group of small non-protein coding RNAs, which are important epigenetic regulators of gene expression [1, 2] and have a role in transcriptional control in a variety of cancers including hepatoblastoma (HB) [3, 4]. miRNA profiling research has identified unique signatures that can be used to classify cancers by determining specific miRNA markers predicting favourable or unfavourable prognoses. Cataloguing the miRNA expression profiles for a large number of different tumour classes may aid in both diagnosis and treatment of cancer [5, 6].

Formalin fixed paraffin embedded (FFPE) samples are a major source of material for HB research. Because of the rare nature of this disease and the limited availability of fresh-frozen tumour samples, it is essential to successfully utilise FFPE samples to obtain high-quality data from these tumours. However, analysis of FFPE material presents obstacles because the process of fixation and embedding, as well as storage time, can negatively impact the quality of RNA isolated from these samples. At a molecular level, modifications occurring from chemical reactions between the fixative and nucleic acids may cause nucleic acid fragmentation and degradation of the RNA [7]. Previous studies have carried out comparative analyses of multiple platforms using non-FFPE material [8, 9]. Other studies have compared platforms for their compatibility with FFPE material but have utilised only one or two platforms, such as microarray with validation using RT-qPCR. Another study used both fresh frozen and FFPE tissue on multiple platforms, however, the sample numbers were very small [10–15]. Studies that have examined HB and miRNAs often used a more targeted approach to investigate candidate miRNAs, rather than performing global profiling of tumour samples [16]. Therefore, the miRNA profile of HB tumours has not been extensively investigated, and a global assessment of the miRNA landscape in HB is lacking.

Here we describe miRNA profiles for 13 HB tumour samples, which we achieved by using a combination of three platforms for miRNA detection. Three of the 13 samples were run using next-generation sequencing (NGS) and a microarray (MA), and a total of 12 of the 13 samples were assessed using NanoString (NS). A comprehensive analysis of the shared miRNA detection across platforms and a comparison of the most highly abundant miRNAs is described.

FFPE material poses challenges to analysis; the most notable being degradation of the sample material. This is an important consideration for generating data and platform selection. Varying amounts of starting material are required for different technologies; for instance, the NGS platform used for analysis required 1 μg of RNA, while the MA required 400 ng, and the NanoString only required 100 ng. These starting amounts can either greatly hinder or enhance the data that can be generated, based on the limited amount of sample available to a researcher. When assessing miRNAs, RNA quality (as determined by the RNA integrity number or RIN, which gives an indication of how intact the total RNA is), may not play as important a role when considering which platform to choose. Our study indicates sample RIN numbers as low as 1.7 produce good quality miRNA data on all the platforms assessed in this study.

## Original purpose

We generated these datasets to describe the miRNA landscape in hepatoblastoma using FFPE samples [17]. In addition, we aimed to describe the strengths and weaknesses of the different platforms: NGS, MA, and NS, for the detection of miRNAs. The level and technical reproducibility for detecting miRNAs in each platform for each sample was investigated and compared between platforms. Further, results were collated to determine the level of shared detection and abundance of specific miRNAs between these three platforms. Hierarchical clustering was performed on the NanoString dataset, which revealed similarities in miRNA profiles in a number of samples, and a unique profiling pattern present in an aggressive HB phenotype [17].

## Sample description

A total of 13 HB tumour samples were evaluated in this study. Three samples (S4, S5 and S6) were analysed with technical replicates on the NGS and MA platforms. S5 and S6 were also analysed with the NS platform (S4 was excluded due to limited sample availability). Further, an additional 10 samples (S7–S16), making a total of 12 HB tumours, were investigated on the NS platform. The age of the patients ranged from 5 months to 10 years 6 months. The samples were a mix of tumour types; the most common being epithelial and fetal, followed by epithelial and mixed fetal embryonal, mixed epithelial mesenchymal and fetal, and finally mixed epithelial mesenchymal and mixed fetal embryonal. One sample (S5) was described histologically as cholangioblastic. Four of the samples contained a mutation in the *CTNNB1* gene (beta-catenin) [18] (Table 1).

**Table 1:** Details of the FFPE hepatoblastoma datasets described in this Data Note

Sample	Patient age	Tumour type + subtype (comments)	Relapse free survival	Overall survival^a^	Pretext at diagnosis	Presence/ location of phosphory-lated -catenin	β-catenin mutation	Platform analysis			Number of miRNAs detected^b^ and GSE accession number		
								NGS	MA	NS	NGS GSE62010	MA GSE62011	NS GSE62017
S4	2 years	Epithelial/mes-enchymal + fetal	3 years 10 months	3 years 10 months (A)	3	cytoplasmic	absent	✓	✓	-	244	83	-
S5	5 months	Epithelial + fetal (cholangioblas-tic)	2 months	7 years (A)	3	cytoplasmic	absent	✓	✓	✓	309	118	299
S6	8 months	Epithelial + fetal (cell clusters, aberrant differentiation)	8 years 11 months	8 years 11 months (A)	3	cytoplasmic	present	✓	✓	✓	321	116	372
S7	10 years 6 months	Epithelial + fetal	1 year 3 months	5 years 4 months (A)	2	cytoplasmic	present	-	-	✓	-	-	216
S8	1 year 7 months	Epithelial + fetal (fibrosis post chemo)	6 years	6 years (A)	2	cytoplasmic + nucleus	absent	-	-	✓	-	-	139
S9	2 years 4 months	Epithelial + fetal	8 years 4 months	8 years 4 months (A)	3	cytoplasmic	absent	-	-	✓	-	-	323
S10	1 year 6 months	Epithelial + fetal/ embryonal	7 years 6 months	7 years 6 months (A)	2	nucleus	present	-	-	✓	-	-	316
S11	9 months	Epithelial + fetal	4 years 10 months	4 years 10 months (A)	3	-	present	-	-	✓	-	-	135
S12	1 year 8 months	Epithelial/mes- enchymal + fetal/ embryonal	9 months	9 months (D)	2	cytoplasmic	absent	-	-	✓	-	-	352
S13	2 years 5 months	Epithelial + fetal	6 years 6 months	6 years 6 months (A)	3	cytoplasmic + nucleus	absent	-	-	✓	-	-	191
S14	7 months	Epithelial + fetal/ embryonal	5 years 3 months	5 years 3 months (A)	2	cytoplasmic	absent	-	-	✓	-	-	411
S15	2 years 2 months	Epithelial + fetal	4 years 6 months	4 years 6 months (A)	2	cytoplasmic	absent	-	-	✓	-	-	163
S16	5 months	Epithelial + fetal	11 years	11 years (A)	4	cytoplasmic + nucleus	absent	-	-	✓	-	-	265

^a^indicates that the overall survival data was last recorded from 2007, (A) and (D) are indicative of status indicator at the time, alive and deceased respectively

^b^indicates S4, S5, and S6 were run in replicate for the NGS and MA platform, therefore the number of miRNAs detected is an average of the replicates

## Platforms used for miRNA quantification

Three platforms were used for miRNA quantification in this study. For the next generation sequencing, 1 μg input RNA was required to construct the small RNA libraries with the TruSeq^®^ Small RNA sample preparation kit (Illumina, San Diego, CA) according to the manufacturers instructions. The Illumina MiSeq platform was used to produce single ended, 50 bp sequenced reads (in FASTQ format). For the microarray platform, 400 ng of input RNA was required. The samples were labelled without amplification with the Affymetrix FlashTag^™^ Biotin HSR RNA Labeling Kit; the labelled samples were hybridised on an Affymetrix^®^ GeneChip^®^ miRNA 3.0 array according to manufacturers guidelines. This chip is able to detect 1733 mature miRNAs based on miRBase version 17. The NanoString platform utilises colour-coded molecular barcodes (probes) to directly hybridise to targets of interest. Single molecule imaging is used to collect highly accurate digital counts of different nucleic acids corresponding to each barcode. We have used Human v2 miRNA Assay Kit for the NanoString platform, which requires 100 ng input RNA and is capable of detecting 800 mature miRNAs based on miRBase version 18. Samples were prepared and analysed according to standard NanoString guidelines for miRNA analysis.

## Data analysis

### Quality assessment of miRNA detection platforms data and post processing

**Fig. 1:**
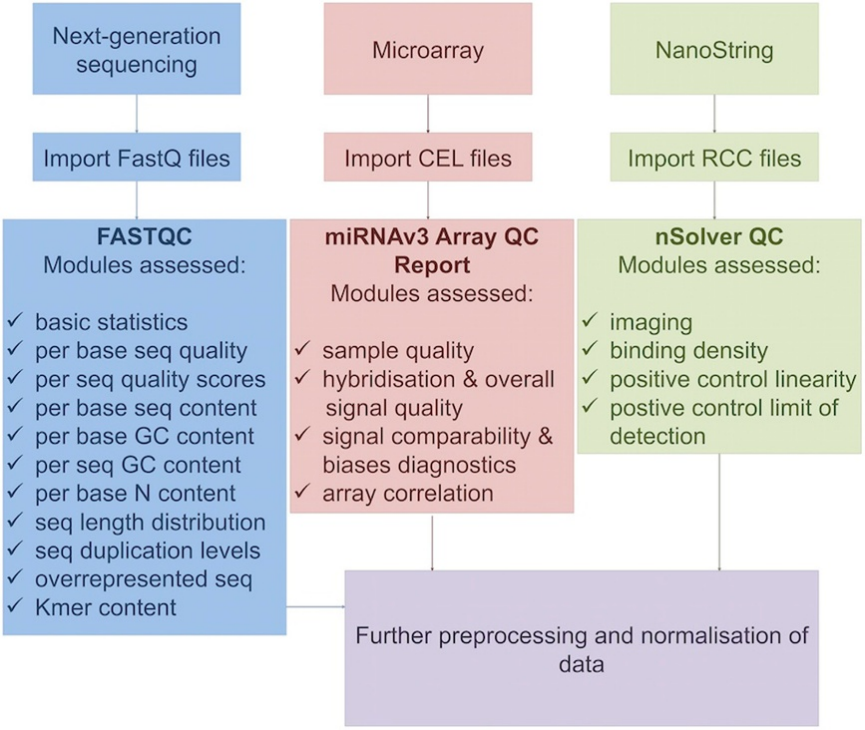
Workflow of the analysis and details of specific strategies for quality assessment of multiple datasets. Check marks corresponding to each quality standard indicate successfully analysed modules for each platform

We briefly describe the processing steps to generate these datasets (Fig. 1). A detailed description of the experimental and analysis steps can be found here [17]. Additionally, to ensure consistent data were achieved using both miRBase versions 17 and 18, the miRNAs were manually checked for inconsistencies, and nomenclature was matched to miRBase version 17. This permitted appropriate comparison of the panel of 800 miRNAs with the microarray and NGS data for further analysis (Additional file 1: Table S1). The proportion of miRNAs mapped for each sample to the total number of identifiable miRNAs from each platform can be found in Additional file 1: Tables S2–S4.

### Next-generation sequencing data

Assessment of the quality of the sequenced reads, quality trimming, and removal of adaptors from the 3’ end of the sequences was performed as previously described [19–22]. The median Phred score of the sequenced bases was > 34 through to the fiftieth sequencing cycle for all the analysed samples. The GC percentage of the samples ranged between 51 and 61. Adapter sequences were removed using Cutadapt [23]. Processed reads were mapped to known miRNAs from the miRBase 17 database using Bowtie1 and miRDeep2 [24, 25]. The number of reads that mapped to an individual miRNA was used to represent its level of expression.

### Microarray data

Raw data in CEL files were normalised using a robust multi-array average (RMA) approach. Median values of each of the probe sets were used to summarise expression values for each microarray chip. Confidently identified miRNAs were determined using a threshold determined by a spike-in control included on the chip (Biob_3). This spike-in has the lowest concentration and represents the limit of detection of the microarray. Probe intensity values below the limit are considered to be background noise and are removed from further analysis. Data quality control was assessed using the miRNAv3 Array QC report. All parameters assessed indicated that high quality data were obtained (Fig. 1).

### NanoString data

Raw data in RCC (reporter code count) files were loaded into nSolver^™^ and used to perform quality assessment and normalisation. A normalisation factor was generated using the geometric mean of the top 100 miRNAs for each sample to offset technical noise. Raw counts were multiplied by the normalisation factor to produce a list of normalised miRNA counts (Fig. 1). Negative controls were included in the expression assays; the limit of detection was calculated by adding 2 SD to the mean of the negative controls (threshold = mean + 2SD). NS data quality was assessed using nSolver^®^ default instructions (version MAN-C0011-03) (NanoString Technologies Inc., nCounter Expression Data Analysis Guide). All samples passed all parameters, with the exception of S6, S7, S14, S15 and S16, which did not pass the positive control limit of detection. Degradation of RNA may have contributed to the low counts of miRNAs being obtained globally in these samples.

### Comparison of miRNA detection and abundance with previous studies

We compared our datasets to several other, relevant published datasets. The first dataset comprised 33 miRNAs identified as differentially expressed between normal tissue and HB by Magrelli et al. [3]. We found that 75.8 % of these differentially expressed miRNAs were also present in our list of 98 miRNAs, (miRNAs detected by at least one sample on all three platforms; significant overlap, *P =* 5.11e-18, hypergeometric test, determined with the reference set of miRNAs as the panel of 800 assessed by NanoString). Commonly detected miRNAs between these studies are reported in Additional file 1: Table S5. When we performed similar analysis with our dataset of 50 miRNAs (miRNAs detected in all of the 12 samples analysed by the NanoString platform), the overlap remained significant (30.3 % overlap, *P =* 1.01e-5, hypergeometric test, determined with the reference set of miRNAs as the panel of 800 assessed by NanoString).

Finally, we compared our data with the GSE21085 hepatoblastoma dataset, which includes non-coding RNA data (analysed on the OSU-CCC MicroRNA Microarray Version 2.0 [condensed version]). We found an overlap of 79 % between our 50 commonly detected miRNAs and GSE21085 (Additional file 1: Table S6). However, this comparison is not completely valid, and should be interpreted with caution: the GSE21085 dataset contains only miRNAs that were known at the time of the particular array design (2005). Additionally, the probes in this array used a selection of pre-miRNAs, while our analysis solely detects the sequences of mature miRNAs.

### Validation by qPCR of miRNAs detected by individual platforms on the remaining material

qPCR is often considered the gold standard method for quantifying RNA expression. We therefore aimed to further validate the miRNAs detected by other platforms. We were able to perform qPCR experiments on only six samples out of the 13 investigated tumours because of the limited availability of RNA from these samples. Validation was performed on five miRNAs (miR-191, miR-95, miR-17, miR-181a, miR-106b), using housekeeping small RNA (RNU6B) as a control. We chose these miRNAs because they have previously been implicated in cancer, and miR-17 and miR-181a are also in the Magrelli dataset [3, 26–29]. NGS and MA platforms provided limited data for these miRNAs (only one sample could be assessed), so we were unable to make a direct comparison of these two platforms with qPCR. However, we were able to compare qPCR quantification with NS, and we observed that NS was better at detecting these five investigated miRNAs in our samples (Additional file 1: Table S7). Our data suggest that in some cases, such as when analysing limited archival FFPE samples, NanoString may be more effective than qPCR for the detection of miRNAs.

## Potential use and application of the data

FFPE material is an important resource: if FFPE samples could be used to their full potential, they will be beneficial to cancer research. These datasets provide a resource describing the strengths and limitations of three platforms used in miRNA detection. These data can serve as a guideline for future research aimed at miRNA profiling, particularly of FFPE samples. Furthermore, 13 HB tumours have been characterised for their miRNA profiles, and since HB is a rare cancer, these datasets can be used in other HB research as a comparison and to supplement further work. Additionally, these data could be used alongside that from other childhood cancers to explore potential relationships and identify related patterns of miRNA expression.

Altered patterns of miRNA expression have previously been identified in HB and several miRNAs have been investigated as predictors of prognosis in patients [3, 30]. miRNA expression levels may help to understand factors contributing to the progression of this disease. For instance, from the datasets described here we identified that S5 had a distinctly altered miRNA expression pattern and clustered differently from the other HB tumours. This particular sample displayed an aggressive phenotype, and had the shortest event-free survival period of the 13 patients. This sort of information will therefore be valuable to establish a better understanding of the relationship between miRNA expression profiles and the severity of HB development in different patients.

## Availability and requirements

The sequencing, microarray and NanoString data have been submitted to the NCBI GEO repository under three different accession numbers (Table 1). All datasets consist of a metadata spreadsheet that provides a summary of the project and the associated files. Downstream analysis using the processed data can be performed with standard computers (4 Gb RAM and 2–4 CPU cores).

The sequencing dataset contains one processed file displaying the raw read counts for each miRNA. This file was used to generate the lists of confidently identified miRNAs using arbitrary thresholds of both ≥ 5 and ≥ 10 reads [17]. Raw Fastq files (total size: 1.07 GB) were included to perform alignment and downstream analysis, if desired by independent researchers.

The microarray dataset contains two files (processed data, in .xlxs format): a file containing all miRNAs from miRBase version 17 and the associated RMA normalised intensity signal for all samples. The second file contains the miRNAs confidently identified using the internal control bioB-3 signal intensity as a threshold for the limit of detection of the array. Raw CEL files comprising the raw signal intensity values of the probes (total size: 17.7 MB) are also included to allow independent processing if desired.

For NanoString, a matrix table with the commercial probe names for the 800 miRNAs is provided in the metadata spreadsheet; the raw data file contains RCC files (total size: 76 KB). These files may be used if alternative normalisation techniques and different detection thresholds are required. Further, two processed files are provided: the first contains the tabulated counts for each miRNA (from miRBase version 18) normalised to the top 100 miRNAs in each sample, and the second file contains only the miRNAs confidently identified after applying a threshold calculated by adding 2SD to the mean of the internal negative controls (threshold = mean + 2SD).

### Project name

Multi-platform microRNA profiling of hepatoblastoma patients using formalin fixed paraffin embedded archival samples

### Operating system(s)

Platform-independent, but UNIX/Linux preferred.

## Availability of supporting data

Datasets supporting the results of this article are available in the NCBI Gene Expression Omnibus archive under accession number GSE62010 (sequencing), GSE62011 (microarray),and GSE62017 (NanoString). Data further supporting this paper can be found in the *GigaScience* Database [31]

## Ethics approval and consent to participate

All clinical data and tumour tissue used in this study were collected with informed consent under ethics approvals from the institutional ethics committees of the participating centres and from the human disability and ethics committee (HDEC) of New Zealand (approval numbers are: CTY/01/10/141 and CTY/01/10/142). The experiment was carried out in accordance with approved guidelines.

## Additional file

Additional file 1: **Supplementary tables, further details of data analysis.** (PDF 197 kb)

## Abbreviations

FFPE: Formalin Fixed Paraffin Embedded
HB: hepatoblastoma
miRNA: microRNA
NGS: Next Generation Sequencing
RMA: Robust Multi-Array Average
