# Supplementary material for: Multi-platform microRNA profiling of hepatoblastoma patients using formalin fixed paraffin embedded archival samples
Source: Gigascience. 2015 Nov 25;4:54. doi: 10.1186/s13742-015-0099-9 (PMC4660849; doi:10.1186/s13742-015-0099-9)
Supplement: Additional file 1: — Supplementary tables, further details of data analysis. (PDF 197 kb) [file 13742_2015_99_MOESM1_ESM.pdf]

Additional Tables S1-S7

Table S1. The miRNA differences documented on miRBase between versions 17 and 18 that were matched manually for analysis

	Accession number	miRBase version 17	miRBase version 18	Changes
1.	MIMAT0000689	hsa-miR-99b	hsa-miR-99b-5p	NAME
2.	MIMAT0000097	hsa-miR-99a	hsa-miR-99a-5p	NAME
3.	MIMAT0000095	hsa-miR-96	hsa-miR-96-5p	NAME
4.	MIMAT0000093	hsa-miR-93	hsa-miR-93-5p	NAME
5.	MIMAT0003218	hsa-miR-92b	hsa-miR-92b-3p	NAME
6.	MIMAT0000092	hsa-miR-92a	hsa-miR-92a-3p	NAME
7.	MIMAT0000441	hsa-miR-9	hsa-miR-9-5p	NAME
8.	MIMAT0004916	hsa-miR-888	hsa-miR-888-5p	NAME
9.	MIMAT0004949	hsa-miR-877	hsa-miR-877-5p	NAME
10.	MIMAT0003888	hsa-miR-766	hsa-miR-766-3p	NAME
11.	MIMAT0004945	hsa-miR-744	hsa-miR-744-5p	NAME
12.	MIMAT0004926	hsa-miR-708	hsa-miR-708-5p	NAME
13.	MIMAT0000252	hsa-miR-7	hsa-miR-7-5p	NAME
14.	MIMAT0004284	hsa-miR-675	hsa-miR-675-5p	NAME
15.	MIMAT0005949	hsa-miR-664	hsa-miR-664-3p	NAME
16.	MIMAT0003326	hsa-miR-663	hsa-miR-663a	NAME
17.	MIMAT0003338	hsa-miR-660	hsa-miR-660-5p	NAME
18.	MIMAT0003337	hsa-miR-659	hsa-miR-659-3p	NAME
19.	MIMAT0003322	hsa-miR-652	hsa-miR-652-3p	NAME
20.	MIMAT0003314	hsa-miR-644	hsa-miR-644a	NAME
21.	MIMAT0018444	hsa-miR-642b	hsa-miR-642b-3p	NAME
22.	MIMAT0003312	hsa-miR-642a	hsa-miR-642a-5p	NAME
23.	MIMAT0004810	hsa-miR-629	hsa-miR-629-5p	NAME
24.	MIMAT0003294	hsa-miR-625	hsa-miR-625-5p	NAME
25.	MIMAT0004807	hsa-miR-624	hsa-miR-624-3p	NAME
26.	MIMAT0004805	hsa-miR-616	hsa-miR-616-3p	NAME
27.	MIMAT0004802	hsa-miR-593	hsa-miR-593-3p	NAME
28.	MIMAT0004799	hsa-miR-589	hsa-miR-589-5p	NAME
29.	MIMAT0003249	hsa-miR-584	hsa-miR-584-5p	NAME
30.	MIMAT0003235	hsa-miR-570	hsa-miR-570-3p	NAME
31.	MIMAT0003225	hsa-miR-561	hsa-miR-561-3p	NAME
32.	MIMAT0003233	hsa-miR-551b	hsa-miR-551b-3p	NAME
33.	MIMAT0018445	hsa-miR-550b	hsa-miR-550b-3p	NAME
34.	MIMAT0004800	hsa-miR-550a	hsa-miR-550a-5p	NAME
35.	MIMAT0015081	hsa-miR-548x	hsa-miR-548x-3p	NAME
36.	MIMAT0015009	hsa-miR-548t	hsa-miR-548t-5p	NAME
37.	MIMAT0005919	hsa-miR-548o	hsa-miR-548o-3p	NAME
38.	MIMAT0005928	hsa-miR-548h	hsa-miR-548h-5p	NAME

39.	MIMAT0005912	hsa-miR-548g	hsa-miR-548g-3p	NAME
40.	MIMAT0018990	hsa-miR-548aj	hsa-miR-548aj-3p	NAME
41.	MIMAT0018972	hsa-miR-548ah	hsa-miR-548ah-5p	NAME
42.	MIMAT0003165	hsa-miR-545	hsa-miR-545-3p	NAME
43.	MIMAT0003164	hsa-miR-544	hsa-miR-544a	NAME
44.	MIMAT0004920	hsa-miR-541	hsa-miR-541-3p	NAME
45.	MIMAT0003163	hsa-miR-539	hsa-miR-539-5p	NAME
46.	MIMAT0002835	hsa-miR-526b	hsa-miR-526b-5p	NAME
47.	MIMAT0002840	hsa-miR-523	hsa-miR-523-3p	NAME
48.	MIMAT0002868	hsa-miR-522	hsa-miR-522-3p	NAME
49.	MIMAT0002829	hsa-miR-519e	hsa-miR-519e-3p	NAME
50.	MIMAT0002869	hsa-miR-519a	hsa-miR-519a-3p	NAME
51.	MIMAT0002842	hsa-miR-518f	hsa-miR-518f-3p	NAME
52.	MIMAT0002861	hsa-miR-518e	hsa-miR-518e-3p	NAME
53.	MIMAT0002848	hsa-miR-518c	hsa-miR-518c-3p	NAME
54.	MIMAT0002866	hsa-miR-517c	hsa-miR-517c-3p	NAME
55.	MIMAT0002852	hsa-miR-517a	hsa-miR-517a-3p	NAME
56.	MIMAT0002859	hsa-miR-516b	hsa-miR-516b-5p	NAME
57.	MIMAT0002883	hsa-miR-514	hsa-miR-514a-3p	NAME
58.	MIMAT0005789	hsa-miR-513c	hsa-miR-513c-5p	NAME
59.	MIMAT0002878	hsa-miR-506	hsa-miR-506-3p	NAME
60.	MIMAT0002876	hsa-miR-505	hsa-miR-505-3p	NAME
61.	MIMAT0004773	hsa-miR-500a	hsa-miR-500a-5p	NAME
62.	MIMAT0019897	hsa-miR-499a-5p	hsa-miR-499b-5p	NAME
63.	MIMAT0019898	hsa-miR-499a-3p	hsa-miR-499b-3p	NAME
64.	MIMAT0002870	hsa-miR-499-5p	hsa-miR-499a-5p	NAME
65.	MIMAT0004772	hsa-miR-499-3p	hsa-miR-499a-3p	NAME
66.	MIMAT0002820	hsa-miR-497	hsa-miR-497-5p	NAME
67.	MIMAT0003161	hsa-miR-493	hsa-miR-493-3p	NAME
68.	MIMAT0004763	hsa-miR-488	hsa-miR-488-3p	NAME
69.	MIMAT0003885	hsa-miR-454	hsa-miR-454-3p	NAME
70.	MIMAT0001635	hsa-miR-452	hsa-miR-452-5p	NAME
71.	MIMAT0001631	hsa-miR-451	hsa-miR-451a	NAME
72.	MIMAT0001545	hsa-miR-450a	hsa-miR-450a-5p	NAME
73.	MIMAT0010251	hsa-miR-449c	hsa-miR-449c-5p	NAME
74.	MIMAT0003327	hsa-miR-449b	hsa-miR-449b-5p	NAME
75.	MIMAT0002814	hsa-miR-432	hsa-miR-432-5p	NAME
76.	MIMAT0001625	hsa-miR-431	hsa-miR-431-5p	NAME
77.	MIMAT0003393	hsa-miR-425	hsa-miR-425-5p	NAME
78.	MIMAT0001341	hsa-miR-424	hsa-miR-424-5p	NAME
79.	MIMAT0003329	hsa-miR-411	hsa-miR-411-5p	NAME
80.	MIMAT0000737	hsa-miR-382	hsa-miR-382-5p	NAME
81.	MIMAT0000735	hsa-miR-380	hsa-miR-380-3p	NAME
82.	MIMAT0000733	hsa-miR-379	hsa-miR-379-5p	NAME
83.	MIMAT0000732	hsa-miR-378	hsa-miR-378a-3p	NAME

84.	MIMAT0000730	hsa-miR-377	hsa-miR-377-3p	NAME
85.	MIMAT0000729	hsa-miR-376a	hsa-miR-376a-3p	NAME
86.	MIMAT0018443	hsa-miR-374c	hsa-miR-374c-5p	NAME
87.	MIMAT0004955	hsa-miR-374b	hsa-miR-374b-5p	NAME
88.	MIMAT0000727	hsa-miR-374a	hsa-miR-374a-5p	NAME
89.	MIMAT0000726	hsa-miR-373	hsa-miR-373-3p	NAME
90.	MIMAT0000719	hsa-miR-367	hsa-miR-367-3p	NAME
91.	MIMAT0000710	hsa-miR-365	hsa-miR-365a-3p	NAME
92.	MIMAT0000707	hsa-miR-363	hsa-miR-363-3p	NAME
93.	MIMAT0000685	hsa-miR-34b*	hsa-miR-34b-5p	NAME
94.	MIMAT0000255	hsa-miR-34a	hsa-miR-34a-5p	NAME
95.	MIMAT0000772	hsa-miR-345	hsa-miR-345-5p	NAME
96.	MIMAT0004692	hsa-miR-340	hsa-miR-340-5p	NAME
97.	MIMAT0003301	hsa-miR-33b	hsa-miR-33b-5p	NAME
98.	MIMAT0000091	hsa-miR-33a	hsa-miR-33a-5p	NAME
99.	MIMAT0000765	hsa-miR-335	hsa-miR-335-5p	NAME
100.	MIMAT0004696	hsa-miR-323-5p	hsa-miR-323a-5p	NAME
101.	MIMAT0000755	hsa-miR-323-3p	hsa-miR-323a-3p	NAME
102.	MIMAT0000090	hsa-miR-32	hsa-miR-32-5p	NAME
103.	MIMAT0000089	hsa-miR-31	hsa-miR-31-5p	NAME
104.	MIMAT0000692	hsa-miR-30e	hsa-miR-30e-5p	NAME
105.	MIMAT0000245	hsa-miR-30d	hsa-miR-30d-5p	NAME
106.	MIMAT0000244	hsa-miR-30c	hsa-miR-30c-5p	NAME
107.	MIMAT0000420	hsa-miR-30b	hsa-miR-30b-5p	NAME
108.	MIMAT0000087	hsa-miR-30a	hsa-miR-30a-5p	NAME
109.	MIMAT0000718	hsa-miR-302d	hsa-miR-302d-3p	NAME
110.	MIMAT0000717	hsa-miR-302c	hsa-miR-302c-3p	NAME
111.	MIMAT0000715	hsa-miR-302b	hsa-miR-302b-3p	NAME
112.	MIMAT0000684	hsa-miR-302a	hsa-miR-302a-3p	NAME
113.	MIMAT0000688	hsa-miR-301a	hsa-miR-301a-3p	NAME
114.	MIMAT0000681	hsa-miR-29c	hsa-miR-29c-3p	NAME
115.	MIMAT0000100	hsa-miR-29b	hsa-miR-29b-3p	NAME
116.	MIMAT0000086	hsa-miR-29a	hsa-miR-29a-3p	NAME
117.	MIMAT0000419	hsa-miR-27b	hsa-miR-27b-3p	NAME
118.	MIMAT0000084	hsa-miR-27a	hsa-miR-27a-3p	NAME
119.	MIMAT0000083	hsa-miR-26b	hsa-miR-26b-5p	NAME
120.	MIMAT0000082	hsa-miR-26a	hsa-miR-26a-5p	NAME
121.	MIMAT0000081	hsa-miR-25	hsa-miR-25-3p	NAME
122.	MIMAT0000080	hsa-miR-24	hsa-miR-24-3p	NAME
123.	MIMAT0000418	hsa-miR-23b	hsa-miR-23b-3p	NAME
124.	MIMAT0000078	hsa-miR-23a	hsa-miR-23a-3p	NAME
125.	MIMAT0000281	hsa-miR-224	hsa-miR-224-5p	NAME
126.	MIMAT0000280	hsa-miR-223	hsa-miR-223-3p	NAME
127.	MIMAT0000279	hsa-miR-222	hsa-miR-222-3p	NAME
128.	MIMAT0000278	hsa-miR-221	hsa-miR-221-3p	NAME

129.	MIMAT0000077	hsa-miR-22	hsa-miR-22-3p	NAME
130.	MIMAT0000275	hsa-miR-218	hsa-miR-218-5p	NAME
131.	MIMAT0000271	hsa-miR-214	hsa-miR-214-3p	NAME
132.	MIMAT0000269	hsa-miR-212	hsa-miR-212-3p	NAME
133.	MIMAT0000268	hsa-miR-211	hsa-miR-211-5p	NAME
134.	MIMAT0000076	hsa-miR-21	hsa-miR-21-5p	NAME
135.	MIMAT0001413	hsa-miR-20b	hsa-miR-20b-5p	NAME
136.	MIMAT0000075	hsa-miR-20a	hsa-miR-20a-5p	NAME
137.	MIMAT0000266	hsa-miR-205	hsa-miR-205-5p	NAME
138.	MIMAT0000265	hsa-miR-204	hsa-miR-204-5p	NAME
139.	MIMAT0002811	hsa-miR-202	hsa-miR-202-3p	NAME
140.	MIMAT0000617	hsa-miR-200c	hsa-miR-200c-3p	NAME
141.	MIMAT0000318	hsa-miR-200b	hsa-miR-200b-3p	NAME
142.	MIMAT0000682	hsa-miR-200a	hsa-miR-200a-3p	NAME
143.	MIMAT0000074	hsa-miR-19b	hsa-miR-19b-3p	NAME
144.	MIMAT0000073	hsa-miR-19a	hsa-miR-19a-3p	NAME
145.	MIMAT0000227	hsa-miR-197	hsa-miR-197-3p	NAME
146.	MIMAT0001080	hsa-miR-196b	hsa-miR-196b-5p	NAME
147.	MIMAT0000226	hsa-miR-196a	hsa-miR-196a-5p	NAME
148.	MIMAT0000461	hsa-miR-195	hsa-miR-195-5p	NAME
149.	MIMAT0000460	hsa-miR-194	hsa-miR-194-5p	NAME
150.	MIMAT0002819	hsa-miR-193b	hsa-miR-193b-3p	NAME
151.	MIMAT0000222	hsa-miR-192	hsa-miR-192-5p	NAME
152.	MIMAT0000440	hsa-miR-191	hsa-miR-191-5p	NAME
153.	MIMAT0000458	hsa-miR-190	hsa-miR-190a	NAME
154.	MIMAT0001412	hsa-miR-18b	hsa-miR-18b-5p	NAME
155.	MIMAT0000072	hsa-miR-18a	hsa-miR-18a-5p	NAME
156.	MIMAT0000262	hsa-miR-187	hsa-miR-187-3p	NAME
157.	MIMAT0000456	hsa-miR-186	hsa-miR-186-5p	NAME
158.	MIMAT0000455	hsa-miR-185	hsa-miR-185-5p	NAME
159.	MIMAT0000261	hsa-miR-183	hsa-miR-183-5p	NAME
160.	MIMAT0000259	hsa-miR-182	hsa-miR-182-5p	NAME
161.	MIMAT0000258	hsa-miR-181c	hsa-miR-181c-5p	NAME
162.	MIMAT0000257	hsa-miR-181b	hsa-miR-181b-5p	NAME
163.	MIMAT0000256	hsa-miR-181a	hsa-miR-181a-5p	NAME
164.	MIMAT0000070	hsa-miR-17	hsa-miR-17-5p	NAME
165.	MIMAT0000069	hsa-miR-16	hsa-miR-16-5p	NAME
166.	MIMAT0000417	hsa-miR-15b	hsa-miR-15b-5p	NAME
167.	MIMAT0000068	hsa-miR-15a	hsa-miR-15a-5p	NAME
168.	MIMAT0000646	hsa-miR-155	hsa-miR-155-5p	NAME
169.	MIMAT0000452	hsa-miR-154	hsa-miR-154-5p	NAME
170.	MIMAT0004697	hsa-miR-151-5p	hsa-miR-151a-5p	NAME
171.	MIMAT0000757	hsa-miR-151-3p	hsa-miR-151a-3p	NAME
172.	MIMAT0000451	hsa-miR-150	hsa-miR-150-5p	NAME
173.	MIMAT0000450	hsa-miR-149	hsa-miR-149-5p	NAME

174.	MIMAT0000243	hsa-miR-148a	hsa-miR-148a-3p	NAME
175.	MIMAT0000251	hsa-miR-147	hsa-miR-147a	NAME
176.	MIMAT0000449	hsa-miR-146a	hsa-miR-146a-5p	NAME
177.	MIMAT0000437	hsa-miR-145	hsa-miR-145-5p	NAME
178.	MIMAT0000436	hsa-miR-144	hsa-miR-144-3p	NAME
179.	MIMAT0000435	hsa-miR-143	hsa-miR-143-3p	NAME
180.	MIMAT0000432	hsa-miR-141	hsa-miR-141-3p	NAME
181.	MIMAT0000430	hsa-miR-138	hsa-miR-138-5p	NAME
182.	MIMAT0000448	hsa-miR-136	hsa-miR-136-5p	NAME
183.	MIMAT0000758	hsa-miR-135b	hsa-miR-135b-5p	NAME
184.	MIMAT0000428	hsa-miR-135a	hsa-miR-135a-5p	NAME
185.	MIMAT0000426	hsa-miR-132	hsa-miR-132-3p	NAME
186.	MIMAT0000691	hsa-miR-130b	hsa-miR-130b-3p	NAME
187.	MIMAT0000425	hsa-miR-130a	hsa-miR-130a-3p	NAME
188.	MIMAT0004605	hsa-miR-129-3p	hsa-miR-129-2-3p	NAME
189.	MIMAT0000445	hsa-miR-126	hsa-miR-126-3p	NAME
190.	MIMAT0000423	hsa-miR-125b	hsa-miR-125b-5p	NAME
191.	MIMAT0000422	hsa-miR-124	hsa-miR-124-3p	NAME
192.	MIMAT0000421	hsa-miR-122	hsa-miR-122-5p	NAME
193.	MIMAT0000254	hsa-miR-10b	hsa-miR-10b-5p	NAME
194.	MIMAT0000253	hsa-miR-10a	hsa-miR-10a-5p	NAME
195.	MIMAT0000680	hsa-miR-106b	hsa-miR-106b-5p	NAME
196.	MIMAT0000103	hsa-miR-106a	hsa-miR-106a-5p	NAME
197.	MIMAT0000102	hsa-miR-105	hsa-miR-105-5p	NAME
198.	MIMAT0000101	hsa-miR-103a	hsa-miR-103a-3p	NAME
199.	MIMAT0000099	hsa-miR-101	hsa-miR-101-3p	NAME
200.	MIMAT0000098	hsa-miR-100	hsa-miR-100-5p	NAME
201.	MIMAT0000415	hsa-let-7i	hsa-let-7i-5p	NAME
202.	MIMAT0000414	hsa-let-7g	hsa-let-7g-5p	NAME
203.	MIMAT0000067	hsa-let-7f	hsa-let-7f-5p	NAME
204.	MIMAT0000066	hsa-let-7e	hsa-let-7e-5p	NAME
205.	MIMAT0000065	hsa-let-7d	hsa-let-7d-5p	NAME
206.	MIMAT0000063	hsa-let-7b	hsa-let-7b-5p	NAME
207.	MIMAT0000062	hsa-let-7a	hsa-let-7a-5p	NAME
208.	MIMAT0002857	hsa-miR-517b	hsa-miR-517b-3p	NAME/SEQUENCE
209.	MIMAT0015073	hsa-miR-3190	hsa-miR-3190-5p	NAME/SEQUENCE
210.	MIMAT0018066	hsa-miR-3647-5p	N/A	DELETED*
211.	MIMAT0018067	hsa-miR-3647-3p	N/A	DELETED*

*These miRNAs have been deleted in miRBase version 18 they were determined to be a fragment of a snoRNA (SNORD111B).

Table S2. The percentage of confidently detected mature miRNAs in each sample mapped to the total of 1733 mature miRNAs able to be detected by the MA.

Sample*	Mapped miRNAs from total in miRBase version 17 for MA (%)
S4	4.8
S5	6.8
S6	6.7

*Indicates the replicates for the samples were averaged to determine confidently detected miRNAs.

Table S3. The percentage of unique alignment and detection of miRNAs using NGS platform

Sample	Number of reads (after adapter removal x 100,000)	Unique alignment efficiency (%)	Mapped miRNAs from total in miRBase version 17 for NGS (5 reads) (%)	Mapped miRNAs from total in miRBase version 17 for NGS (10 reads) (%)
S4A	8.3	31.0	17.7	15.0
S4B	5.5	29.0	15.8	13.2
S5A	9.1	60.0	23.5	19.9
S5B	4.4	59.0	19.6	16.1
S6A	10.0	55.0	22.2	18.9
S6B	8.6	56.0	21.0	18.2

Table S4. The percentage of confidently detected mature miRNAs with NS platform in each sample (mapped to the total panel of 800 mature miRNAs in NS)

Sample	Mapped miRNAs from total panel for NS (%)
S5	37.4
S6	46.5
S7	27.0
S8	17.4
S9	40.4
S10	39.5
S11	16.9
S12	44.0
S13	23.9
S14	51.4
S15	20.4
S16	33.13

Table S5. Commonly detected miRNAs between Leichter et al and Magrelli et al 2009.

miRNAs common between Leichter et al/ Magrelli et al
hsa-miR-455-3p
hsa-342-3p
hsa-miR-30b
hsa-miR-30a
hsa-miR-221
hsa-miR-214
hsa-miR-20a
hsa-miR-199a-5p
hsa-miR-199a-3p
hsa-miR-195
hsa-miR-194
hsa-miR-192
hsa-miR-181a
hsa-miR-17
hsa-miR-15b
hsa-miR-15a
hsa-miR-155
hsa-miR-150
hsa-miR-145
hsa-miR-143
hsa-miR-140-3p
hsa-miR-125a-5p
hsa-miR-10a
hsa-miR-106a
hsa-let-7a

Table S6. The commonly detected miRNAs from between Leichter et al. and the GSE21085 dataset

miRNAs* common between GSE21085 dataset and Leichter et al.
hsa-let-7a
hsa-let-7b
hsa-let-7c
hsa-let-7d
hsa-let-7e
hsa-let-7f
hsa-let-7g
hsa-let-7i
hsa-miR-100
hsa-miR-106a
hsa-miR-106b
hsa-miR-122
hsa-miR-125b
hsa-miR-126
hsa-miR-127
hsa-miR-140
hsa-miR-144
hsa-miR-145
hsa-miR-148b
hsa-miR-15
hsa-miR-17
hsa-miR-181
hsa-miR-191
hsa-miR-192
hsa-miR-195
hsa-miR-199a
hsa-miR-199b
hsa-miR-22
hsa-miR-222
hsa-miR-23a
hsa-miR-23b
hsa-miR-25
hsa-miR-26a
hsa-miR-27b
hsa-miR-29a
hsa-miR-29c
hsa-miR-302d
hsa-miR-337
hsa-miR-34a

hsa-miR-95
hsa-miR-99a

* indicates that miRNAs assessed were both pre-miRNAs (GSE21085 dataset) and mature miRNAs (Leichter et al. dataset), therefore the commonly detected miRNAs were determined from a pre-miRNA and were given a neutral nomenclature without the addition of 3p and 5p suffixes because this is unable to be defined. This caveat has been discussed in the manuscript.

Table S7. qPCR Validation of five miRNAs in 6 HB samples.
An ✗ indicates that the miRNA has not been successfully detected while a ✓ indicates the miRNA was detected above the previously determined threshold of confidently identified.

**Sample / validated assays**		**Platforms assessed**		
	**qPCR**	**NS**	**NGS**	**MA**
**S6**				
RNU6B*	✓	N/A	N/A	N/A
hsa-miR-191	✓	✓	✓	✓
hsa-miR-95	✓	✓	✓	✗
hsa-miR-17	✓	✓	✓	✓
hsa-miR-181a	✓	✓	✓	✓
hsa-miR-106b	✓	✓	✓	✓
**S7**				
RNU6B*	✗	N/A	N/A	
hsa-miR-191	✗	✓		
hsa-miR-95	✗	✓		
hsa-miR-17	✗	✓		
hsa-miR-181a	✓	✓		
hsa-miR-106b	✗	✓		
**S8**				
RNU6B*	✗	N/A		
hsa-miR-191	✗	✓		
hsa-miR-95	✗	✓		
hsa-miR-17	✗	✓		
hsa-miR-181a	✗	✓		
hsa-miR-106b	✗	✓		

**S10**			
RNU6B*	✗	N/A	
hsa-miR-191	✗	✓	
hsa-miR-95	✗	✓	
hsa-miR-17	✗	✓	
hsa-miR-181a	✗	✓	
hsa-miR-106b	✗	✓	
**S11**			
RNU6B*	✓	N/A	
hsa-miR-191	✓	✓	
hsa-miR-95	✓	✓	
hsa-miR-17	✓	✓	
hsa-miR-181a	✓	✓	N/A
hsa-miR-106b	✓	✓	
**S15**			
RNU6B*	✗	N/A	
hsa-miR-191	✗	✓	
hsa-miR-95	✗	✓	
hsa-miR-17	✗	✓	
hsa-miR-181a	✗	✓	
hsa-miR-106b	✗	✓	

*Indicates that RNU6B is a small RNA that was used as a housekeeping gene in qPCR experiments therefore its detection of RNU6B is not applicable for other platforms.

Out of the 3 samples investigated for all three platforms, only one was available for further analysis. In addition, we used five more samples for which only NS data was available. Therefore a comparison between only NS and qPCR can be made.

N/A= means it was not assessed by these platforms.
